# Effect of Carbon Content on Friction and Wear Properties of Copper Matrix Composites at High Speed Current-Carrying

**DOI:** 10.3390/ma12182881

**Published:** 2019-09-06

**Authors:** Zhenghai Yang, Yuexin Ge, Xu Zhang, Bao Shangguan, Yongzhen Zhang, Junwei Zhang

**Affiliations:** 1 National Laboratory of High-end Bearing Tribology Technology and Application, School of Materials Science and Engineering, Henan University of Science and Technology, Luoyang 471023, China (Y.G.) (X.Z.) (B.S.) (Y.Z.) (J.Z.); 2 State Key Laboratory of Solid Lubrication, Lanzhou Institute of Chemical Physics, Chinese Academy of Sciences, Lanzhou 730000, China

**Keywords:** graphite content, copper matrix composites, current-carrying friction, friction and wear properties, current-carrying properties

## Abstract

The copper matrix composites were prepared by spark plasma sintering (SPS). The current-carrying friction and wear tests were carried out on a self-made HST-100 high-speed current-carrying friction and wear tester, and the effect of the graphite content on the current-carrying friction and wear properties of the composite material was studied. The results show that with an increase in graphite content, the average friction coefficient and wear rate of the two materials decreased significantly, the fluctuation amplitude of the friction coefficient was also significantly reduced, and the average friction coefficient of copper-coated graphite composite with graphite content of 10 wt.% was 0.100; when the graphite content was the same and more than 5.0 wt.%, the average friction coefficient and wear rate of copper–graphite composites were slightly higher than copper–copper-coated graphite composites; the current-carrying efficiency and current-carrying stability of the copper matrix composite were obviously higher than that of copper material; there was a mechanical wear area and arc erosion area on the wear surface of the composites, with the increase in graphite content, the adherence and the tear of the mechanical wear area weakened, the rolling, plastic deformation increased, and the surface roughness decreased obviously. The surface roughness of the wear surface of copper–copper-coated graphite composites with graphite content of 10 wt.% was 3.17 μm. The forms of arc erosion included melting and splashing, and were mainly distributed in the friction exit area.

## 1. Introduction

The current-carrying friction pair is a friction pair with a conduction current function [1], and its contact surface requires both a current conduction capability and friction and wear performance. Current-carrying friction is a process of coupling the frictional contact of a rough surface with conductive contact, accompanied by arc discharge and other phenomena [2,3]. The service behavior of different current-carrying friction materials is different, and the macroscopic performance is different [4,5]. With developments in science and technology, the service conditions of the current-carrying friction pair continue to expand, for example, the relative sliding speed continues to increase.

Copper matrix composites are commonly used as current-carrying friction materials, and the copper matrix of the three-dimensional network structure satisfies the requirements of electrical conductivity, enhances the phase lubrication, and improves the friction and wear performance [6,7,8,9]. Since the gas oxide generated after oxidation does not affect the subsequent conduction, and can prevent the oxidation of copper [10], sp2 hybrid carbon materials, such as graphite [11], carbon fiber [12], or carbon nanotube [13] are common reinforcing phases. The common preparation method for copper–carbon composite materials is powder metallurgy, which includes the processes of hot-pressing sintering [14] and spark plasma sintering (SPS) [15].

There are many previous studies on copper–carbon (sp2 hybrid) composites. Ma W. et al. [16] studied the friction and wear properties of copper–graphite composites with 2024 aluminum alloy, AZ91D magnesium alloy, and Ti6Al4V titanium alloy, the results show that the copper–graphite composite is a good self-lubricating material, and the sliding speed affects the friction interface, which affects the friction and wear mechanism. Pietrzak et al. [17] studied the effects of carbon morphology on the material organization and thermal properties of copper–carbon composites and the lubricating behavior and conductivity of the graphite films at low speed. The studies of Gao Qiang et al. [18] show that the conductivity of the copper–graphite composite prepared by pressing-sintering has the best carbon content. The results of Ge Yuexin and other studies [19] show that the copper–graphite composites prepared by the SPS process at 780 °C have the best current-carrying friction properties. The studies of He Dahai and Fan Yi et al [20,21] show that natural flake graphite can form a complete and stable lubricating graphite film on the friction surface of the copper matrix composite, which is beneficial for preventing the friction material from sticking to the dual and improving the friction stability. According to Feng Yi et al. [22], a reasonable addition of the WS2 to the copper–graphite composite can significantly improve the wear resistance of the material without increasing the power loss. The studies of Xu Wei et al [23] show that the friction coefficient and wear rate of the Cu-graphite composites decrease with an increase in graphite content, and increases with an increase in the current density, and the main wear mechanism is arc erosion and adhesive wear. Previous studies have shown that the content of graphite has an important influence on the properties of the material and the friction and wear behavior. However, most of these studies focused on the condition of low speed (relative sliding velocity is less than 1 m/s), and with the expansion of the service conditions of the current-carrying friction pair, it is necessary to explore their service performance at high speed.

Therefore, in this study, pure copper, copper–graphite composites, and copper–copper-coated graphite composites were prepared by spark plasma sintering (SPS) using pure copper powder, natural graphite powder, and copper-coated graphite powder as raw materials, the effects of carbon content on current-carrying friction and wear properties of copper-based materials under high-speed sliding conditions were studied.

## 2. Experimental Materials and Methods

### 2.1. Material Preparation

The copper-based materials were prepared by SPS with pin samples for testing. The raw materials were electrolytic pure copper powder with a purity of more than 99%, copper-coated graphite powder, and natural graphite powder. The particle size of the powder was 75 μm, and the graphite content in copper-coated graphite powder was 50 wt.%. The preparation process of the material was as follows. Powder was mixed on a V-type mixer for 18 h and SPS process under vacuum condition. The sintering pressure was 30 MPa, the sintering temperature was 780 °C, the heating rate was 100 °C/s, and the holding time was 5 min. after sintering, the sintered material was cooled with the furnace, and the sintered material was cut into ϕ10 mm pin samples by wire cutting. Copper–graphite composites and copper-coated graphite composites with graphite content of 0, 2.5 wt.%, 5 wt.%, 7.5 wt.%, 10 wt.%, and 12.5 wt.% were prepared. However, due to the poor properties of 12.5 wt.% copper matrix materials, they did not participate in the subsequent test.

### 2.2. Performance Testing

**Figure 1:**
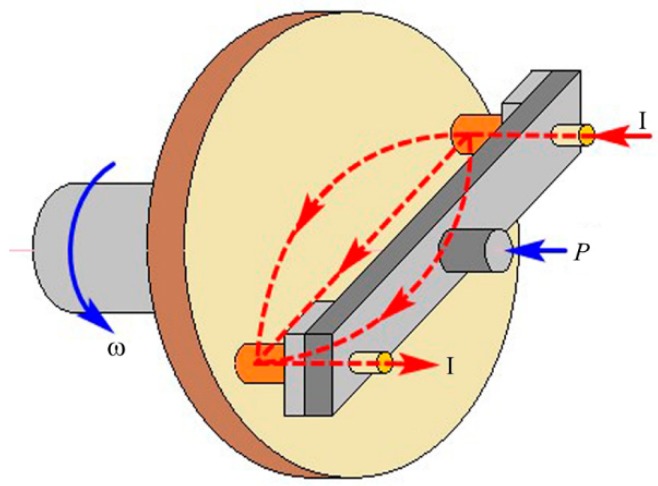
Basic schematic of the HST-100 high speed pin-on-disc tribo-tester.

The friction and wear tests were carried out on the self-made HST-100 high-speed friction testing machine (see Figure 1). The friction pair was a pin-disc type, the current flowed out of one pin sample, through the disc sample, and back from the other pin sample, and the two pin samples were the same material. The sample material was QCr0.5, the positive pressure 70 N, current 100 A, relative sliding speed 20 m/s, test time 30 s. Before the test, the sample was polished with 800# sandpaper and pre-ground on the testing machine without electricity for 10 min with a speed of 5m/s, each experiment was repeated three times, and the results were averaged.

The wear surface was observed by a JSM-5610LV scanning electron microscope (SEM, JEDL, Tokyo, Japan) with energy dispersive spectrometer(EDS), the conductivity was measured by a Sigma2008B/C digital eddy current metal conductometer (Shanghai GaoZhi Precision Instrument Co., Ltd, Shanghai, China), the density was measured by the drainage method, the hardness was measured by a HV-1000 microhardness tester (Laizhou Huayin Testing Instrument Co., Ltd, Laizhou, China), and the surface roughness was measured by a nano focus three-dimensional topography instrument (Nanofocus AG, Oberhausen, Germany).

In the current-carrying friction and wear test, the friction and wear properties were evaluated by friction coefficient and mass wear rate. Current-carrying performance was evaluated by current-carrying efficiency and current-carrying stability. The current-carrying efficiency represented the ability of the friction pair to transmit the current during the service process, which was the ratio of the average value of the actual current to the given current in the service process. The current-carrying stability represented the fluctuation of conduction current during the service of friction pair. The calculation formula is as follows:

$$\delta =\left(1-\frac{\sigma }{\bar{I_{i}}}\right)\times 100\%,$$

where δ is the current-carrying stability parameter, %, dimensionless, the larger the value, the higher the current-carrying stability; σ is the standard deviation for current, A; $\bar{I_{i}}$ is the average value of the actual current in the course of the test., A.

## 3. Results and Analysis

### 3.1. Microstructure and Properties of Composites

**Figure 2:**
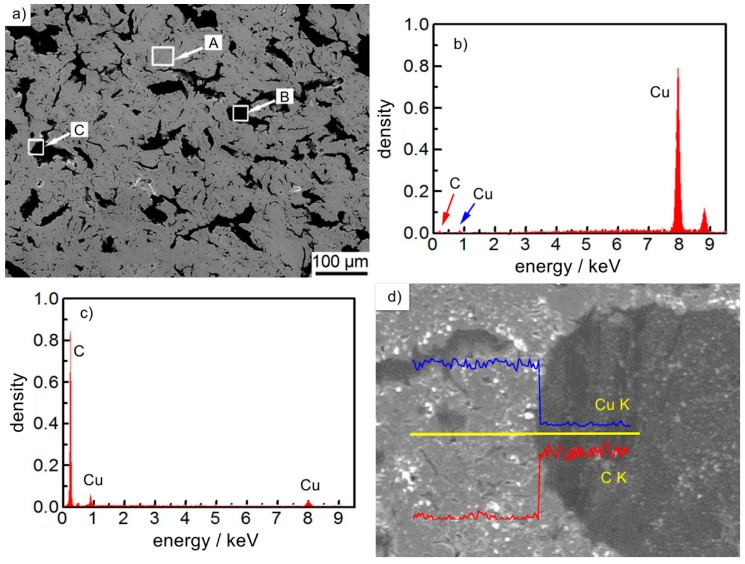
The scanning electron microscope (SEM) and energy dispersive spectrometer (EDS) of Cu-7.5wt% graphite composite materials: (**a**) the SEM of diagram organization, (**b**) the energy spectrum diagram of region A, (**c**) the energy spectrum diagram of region B, (**d**) the line energy spectrum diagram of region C.

Figure 2 shows the microstructure and energy spectra of Cu-7.5 wt% graphite composites, Figure 2a shows the SEM picture of the material cross-section. Figure 2b shows the energy spectra diagram of region A in Figure 2a, Figure 2c shows the energy spectra diagram of region B in Figure 2a, and Figure 2d is the scanning diagram of region C in Figure 2a. It can be seen from the figure that the gray area in Figure 2a (region A) is Cu material (Figure 2b), black area is graphite material (Figure 2c), graphite was uniformly distributed in the copper matrix; the interface between graphite and copper was close and there was no obvious pore. The results of line scanning show that copper and carbon jumped at the interface, the peak value at the boundary changed obviously, there was no overlapping region, and the content was stable after jumping. Because Cu and C were completely insoluble elements, the interface existed by mechanical bonding, which indicates that the interface of the prepared material was close and there was no obvious gap.

**Figure 3:**
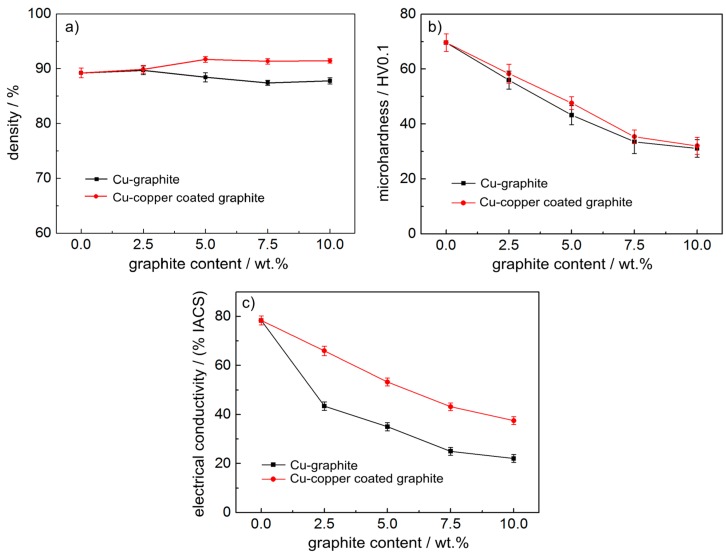
The material density, Vickers hardness, and conductivity of composite materials: (**a**) density, (**b**) Vickers hardness, (**c**) conductivity.

Figure 3 shows the density, Vickers hardness, and conductivity of copper–carbon composites with different carbon content. As shown in Figure 3a, with an increase in graphite content, the density of the copper–graphite composites and copper-coated graphite composites fluctuated slightly at about 90%, and the density of copper-coated graphite composites was slightly better. It can be seen from Figure 3b that, with an increase in graphite content, the hardness of the composites decreased sharply, and the area was gentle after the graphite content was 7.5 wt.%. The properties of copper-coated graphite composites were slightly better than those of copper–graphite composites with the same carbon content. It can be seen from Figure 3c that the conductivity of the composites decreased sharply with an increase in graphite content, and the conductivity of copper-coated graphite composites was obviously better than that of copper–graphite composites with the same graphite content.

For copper–graphite composites, when graphite components were added to the copper matrix, copper and graphite were deformed during spark plasma sintering. The bonding between copper and copper belongs to metallurgical bonding, graphite and copper are mechanically bonded, and the interface between copper and graphite is electroplated in copper-coated graphite composites. There was no significant difference in density and hardness between the two kinds of materials. The results show that the interface between copper and graphite in the copper–graphite composites interacted well in the sintering process of SPS. The conductivity of copper in the two kinds of materials was much better than that of graphite, so with the increase in graphite content, the conductivity of the materials decreased obviously. At the same time, because the electroplating interface of copper-coated graphite was better than that of copper and graphite sintered by SPS process, the conductivity of copper–graphite composites was not as good as that of copper-coated graphite composites under the same other conditions.

### 3.2. Effect of Carbon Content on the Friction Coefficient and Wear Rate of Copper-Based Materials

**Figure 4:**
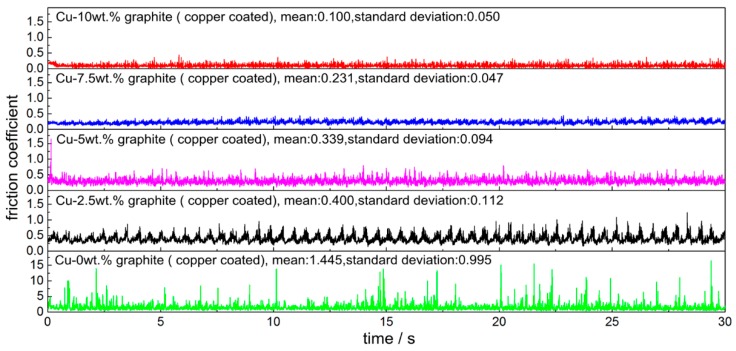
The curve of dynamic current-carrying friction coefficient.

Figure 4 shows the dynamic friction coefficient of copper-coated graphite composite and QCr0.5. It can be seen from the diagram that the friction coefficient fluctuated obviously in the process of each test, but the whole was in a stable state, which shows that the pre-grinding effect was good and the running-in was completed quickly with the pair during the test. When the graphite content was 0, the average friction coefficient was 1.445, the variance of the friction coefficient was 0.995, and the maximum friction coefficient was 17.5. That is to say, the cold welding and tearing process between the pin and disk led to the sharp increase in friction coefficient. When graphite existed in the composite, the average value and variance of friction coefficient decreased sharply. With the increase in graphite content, the average value and variance of the friction coefficient decreased. That is to say, the friction process became more and more stable. When the graphite content reached 10 wt.%, the friction coefficient was only 0.100.

**Figure 5:**
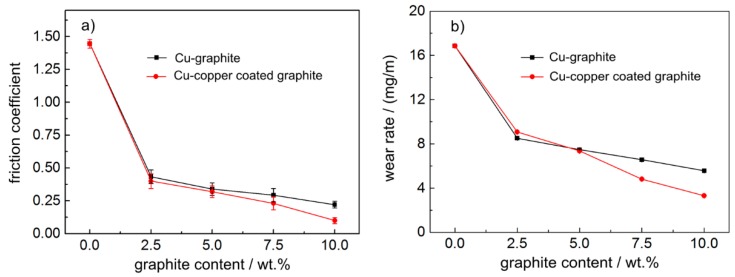
The friction and wear performance curve of composites versus QCr0.5: (**a**) friction coefficient, (**b**) wear rate.

Figure 5 shows the friction and wear properties of composites with different graphite content. Figure 5 shows that the average friction coefficient and wear rate of the two materials decreased significantly with an increase in carbon content. When the carbon content was the same and lower than 5.0 wt.%, the average friction coefficient and wear rate of the two materials were not significantly different, but when the carbon content was the same and higher than 5.0 wt.%, the friction coefficient and wear rate of copper–graphite composites were slightly higher than those of copper-coated graphite composites.

### 3.3. Effect of Graphite Content on Current-carrying Efficiency and Current-carrying Stability of Materials

**Figure 6:**
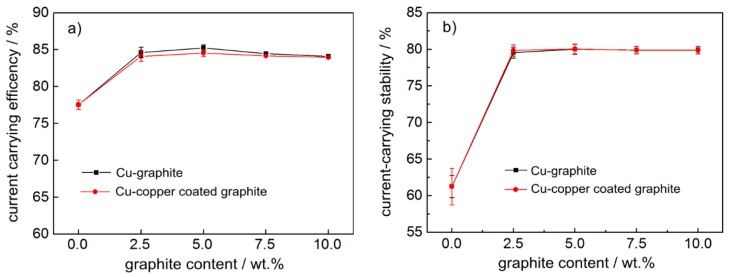
The variation curve of current-carrying performance: (**a**) current-carrying efficiency, (**b**) current-carrying stability.

Figure 6 shows the variation curve of current-carrying performance of copper-based materials with different graphite content. It can be seen from Figure 6 that the current-carrying efficiency and current-carrying stability of pure copper materials in powder metallurgy were very low, only about 77% and 60%, respectively, when graphite was added, the current-carrying efficiency and current-carrying stability obviously improved, reaching 84% and 80%, respectively, and the numerical values did not change much with the increase in graphite content. When the lubrication phase content was the same, the electrical conductivity of copper-coated graphite composites was slightly worse than that of copper–graphite composites.

### 3.4. Current-Carrying Friction Behavior of Composites

**Figure 7:**
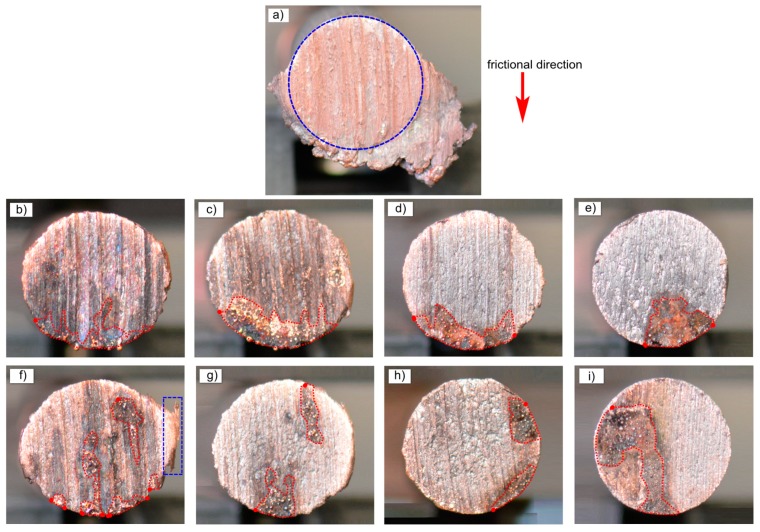
Morphology pictures of worn surface: (**a**) Cu, (**b**) Cu-copper coated graphite (2.5 wt.% C), (**c**) Cu-copper coated graphite (5 wt.% C), (**d**) Cu-copper coated graphite (7.5 wt.% C), (**e**) Cu-copper coated graphite (10 wt.% C), (**f**) Cu-2.5 wt.% graphite, (**g**) Cu-5wt.% graphite, (**h**) Cu-7.5 wt.% graphite, (**i**) Cu-10 wt.% graphite.

Figure 7 is a macro picture of the worn surface of copper-based materials after current-carrying friction. It can be seen from Figure 7a that the friction surface mechanical wear of pure copper material in powder metallurgy was serious, the position of pin sample is shown in the circle, and a large number of friction attachments appeared outside the circle. It can be seen from Figure 7b–e and Figure 7f–i, respectively, that the wear surface could be divided into two parts: the mechanical wear area and the arc erosion area (red dotted line marked area in the figure). At the same time, friction attachments also appeared in the blue rectangular region in Figure 7f. From the distribution area of arc erosion, the arc erosion area was mainly distributed in the current-carrying friction exit area. For copper-coated graphite composites, this law is very stable. For copper–graphite composites, there were electric arc ablation areas in the exit area, and there was also serious arc erosion in other regions.

**Figure 8:**
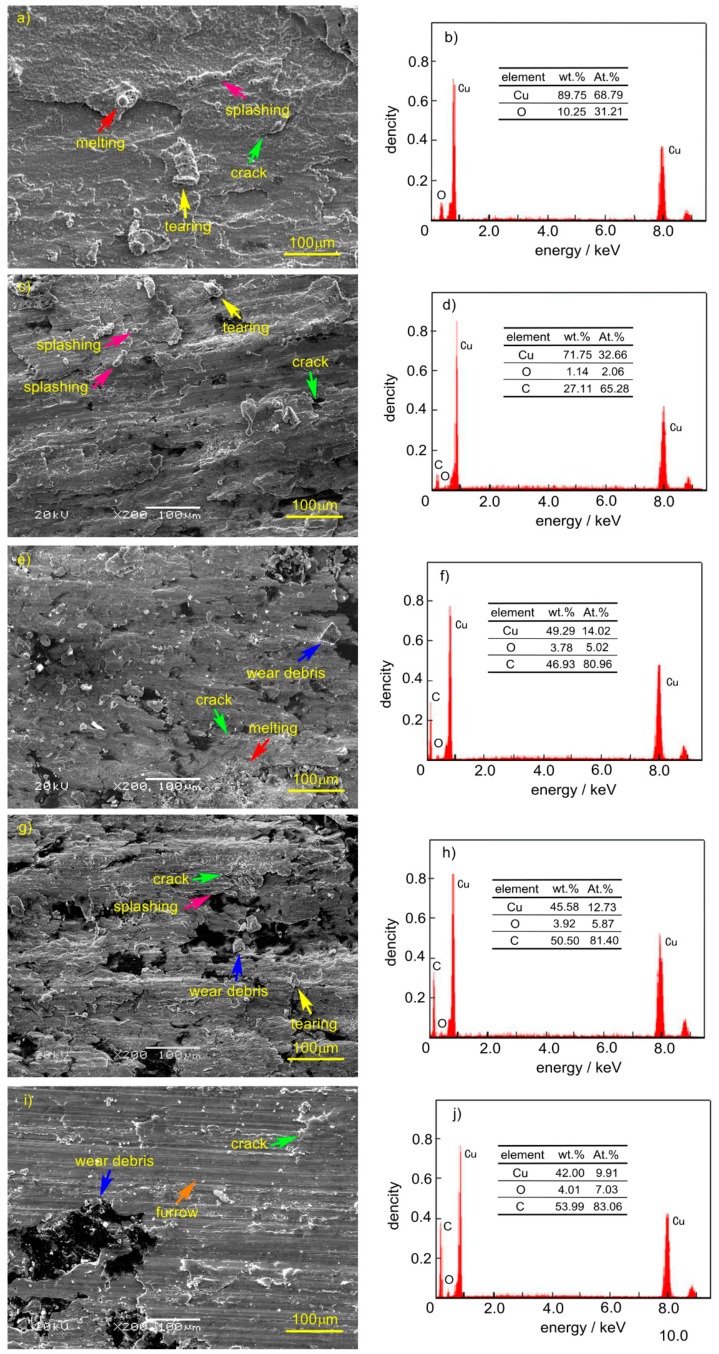
The SEM and EDS pictures of mechanical wear areas of copper matrix composites: (**a**) Cu, (**b**) the energy spectrum diagram of Figure 8a, (**c**) Cu-copper coated graphite (2.5wt.% C), (**d**) the energy spectrum diagram of Figure 8c, (**e**) Cu-copper coated graphite (5 wt.% C), (**f**) the energy spectrum diagram of Figure 8e, (**g**) Cu-copper coated graphite (7.5 wt.% C), (**h**) the energy spectrum diagram of Figure 8g, (**i**) Cu-copper coated graphite (10 wt.% C), (**j**) the energy spectrum diagram of Figure 8i.

Figure 8 shows the SEM and EDS pictures of the mechanical wear area of copper matrix composites prepared by the SPS process. It can be seen from the diagram that the wear formed in current-carrying friction included adhesion, tearing, furrow, melting, splashing, plastic deformation, and oxidation. Among these, spatter marks included spherical and long strip shapes, the spherical diameter was relatively small, and the long strip size was larger. There were two kinds of cracks on the wear surface, one was the tear crack after adhesion (Figure 8a), the other was the crack after rolling deformation, which appeared beyond plasticity (Figure 8i). Oxygen elements existed on all wear surfaces, and the oxygen content on the wear surface of pure copper was obviously higher than that on the surface of copper–graphite composites. The content of carbon on the surface increased with the increase in graphite content. There was no carbon on the worn surface without graphite. When the graphite content of the composite was 2.5 wt.%, the carbon content on the wear surface was 27.11 wt.%. After the graphite content of composite reached 5 wt.%, the carbon content on the wear surface was close at first, then more than 50 wt.%; although it increased, the range is small. When the graphite content in the composite reached 5 wt.%, the graphite content was stable. It can also be seen from the diagram that the trace scale of tear became smaller with the increase in carbon content in the material, and the degree of furrow and plastic deformation increased gradually with the increase in carbon content in the material.

Table 1 shows the average surface roughness of the main area of mechanical wear in Figure 8. It can be seen from the table that the roughness of the mechanical wear surface decreased obviously with the increase in graphite content. The surface roughness of the copper-coated graphite composite was smaller than that of the copper–graphite surface, and reached the minimum when the graphite content reached 10 wt.%, which was 3.17 μm.

**Figure 9:**
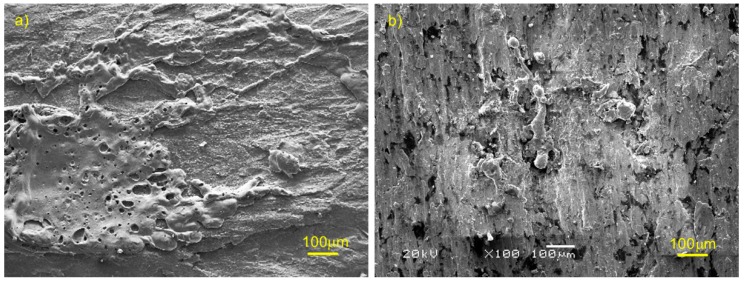
Arc erosion of materials: (**a**) Cu, (**b**) Cu-7.5 wt.% graphite composite.

Figure 9a shows the arc erosion morphology of the Cu material, from which the traces of melting and splashing can be seen, and the melting marks of the material were flat and covered on the matrix. Figure 9b shows the arc erosion morphology of Cu-7.5 wt.% graphite composites. It can be seen from the diagram that the molten materials gathered together, this is because copper and graphite were not wetting at all, and the molten liquid aggregated and solidified under the action of surface tension. The spatter trace was approximately strip-shaped and approximately spherically-shaped, when the amount of molten material splashed was greater, the space flight time was short, and the strip was formed under the action of flight; when the splashing molten material was less, it was spherical and cooled under the action of surface tension, and maintained the approximate spherical shape when it fell on the surface.

## 4. Discussions

The contact of current-carrying friction is actually the contact of the rough surface, and friction and wear (mainly including adhesion, tear, plough, rolling, plastic deformation), contact conduction current, arc discharge, oxidation, and other processed appeared in the process of current-carrying friction.

For the contact and friction and wear process of rough surfaces, the contact pressure and surface composition are key factors from the point of view of a single micro-convex peak. It is closely related to the number and stability of contact micro-convex peaks from the point of view of the whole friction surface. The results show that the smaller the roughness of the friction surface is, the more micro-convex peaks are involved in contact, and the smaller the positive pressure on a single micro-convex peak is when the total pressure is unchanged, the smaller the area of adhesion is and the smaller the tear trace is, and the smaller the deterioration in surface quality caused by the tear and the adhesion at the early stage. At the subsequent friction process, the smaller the impact, the smaller the vibration, the more stable the running of the friction pair (The fluctuation of friction coefficient in Figure 4 illustrates this), the adhesion of the material, and the smaller the tear trace is. With an increase in graphite content, the more graphite may be distributed on the friction surface, the more it can prevent the occurrence of adhesion, and it can be transformed into the compaction of hard point on soft matrix and the process of plastic deformation. Therefore, with the increase in graphite content, the surface roughness decreases, the adhesion and the tear trace decreases, and the degree of plastic deformation caused by rolling surface becomes greater. However, too much graphite can cause the strength of the composites to be too low (so the test of 12.5 wt.% graphite content was not completed.).

For the conductive contact process, the material and size of the spot are the key factors from the point of view of a single contact conductive spot (α spot), and the dynamic change process of the quantity and quality of the spot in the friction process is the key factor from the point of view of the whole contact surface (the contact resistance of the contact surface decreases as the number of α spots increases, and the number of α spots exceeds a certain value, and tends to be stable). Therefore, the current-carrying efficiency and current-carrying stability of friction pairs are mainly related to the quality and quantity of spots and their variation with time. When the friction pair fluctuates greatly during the operation of the friction pair (Cu material in Figure 4), the quality and quantity of the α spots change dramatically with time, and the current-carrying efficiency and current-carrying stability of the friction pair are poor; when the friction pair is relatively stable (Cu matrix composites in Figure 4), although the quality and quantity of α spots change, the current-carrying efficiency and current-carrying stability of the friction pair tend to be stable.

The arc discharge process occurs in the process of contact formation and failure of the friction pair, and the stability of friction pair operation is an important influencing factor. With the increase in graphite content, the friction pair runs more and more smoothly (the variance of the friction coefficient in Figure 4), which can suppress the harm caused by electric arc discharge. However, due to the randomness and dynamics of the arc discharge, this effect is not obvious in terms of the arc ablation area.

During the friction process of the composite, the oxide of copper is retained on the worn surface, and the oxide of carbon is the gas. The oxygen content on the surface of the composite is measured, which directly reflects the oxidation of copper on the surface. The carbon element is more alive than the copper element, therefore, the existence of carbon element on the wear surface of copper–graphite composite suppresses the oxidation of copper.

To sum up, in the mechanical wear area, graphite falls off on the surface, smeared on the wear surface through interlaminar deformation, and a reasonable thickness of graphite film on the friction surface is the key to obtain good performance materials. Combining the results of the EDS analysis in Figure 8 and the friction and wear properties of the composites, the authors believe that when the graphite content exceeds 5 wt.%, the friction surface forms a continuous graphite film, and when the graphite content reaches 10 wt.%, the effect of graphite film is the best.

## 5. Conclusions

The copper matrix composites were prepared by the spark plasma sintering process, matched with QCr0.5. The current-carrying friction test was carried out on the self-made HST-100 high-speed friction tester to study the effect of graphite content on the current-carrying friction properties of copper-based materials. The results show that:

(1) The interface bonding of copper matrix composites was close and there was no obvious gap. The hardness and conductivity of the copper matrix composites decreased with the increase in graphite content;

(2) With the increase in graphite content, the average friction coefficient and wear rate of the two materials decreased significantly, the fluctuation amplitude of the friction coefficient also significantly reduced, and the average friction coefficient of the copper–copper-coated graphite composite with a graphite content of 10wt.% was 0.100. When the graphite content was the same and more than 5.0 wt.%, the average friction coefficient and wear rate of copper–graphite composites were slightly higher than those of copper-coated graphite composites. The current-carrying efficiency and current-carrying stability of the copper matrix composite were obviously higher than that of the copper material, which reached 84% and 80% respectively;

(3) There was mechanical wear area and arc erosion area on the wear surface of the composites. The machinery wear formed included adhesion, tearing, furrow, roller compaction, and plastic deformation. With the increase in graphite content, adhesion, tear weakening, rolling, and plastic deformation increased; with the increase in graphite content, the roughness of the mechanical wear surface decreased obviously. The surface roughness of the copper–copper-coated graphite composite surface was smaller than that of the copper–graphite surface. When the graphite content reached 10 wt.%, it reached the minimum, which was 3.17 μm. The forms of arc erosion mainly included melting and splashing, mainly distributed in the friction exit area, and spatter traces included spherical and strip-shaped.

## Figures and Tables

**Table 1:** Surface roughness corresponding to Figure 8 (Ra/μm).

**Graphite Content**	0	2.5 wt.%	5 wt.%	7.5 wt.%	10 wt.%
**Cu-Graphite**	13.02	9.25	8.57	7.16	6.11
**Cu-copper Coated Graphite**	-	8.07	7.67	5.24	3.17

## References

[1] Wang Z.Y., Guo F.Y., Chen Z.H., Tang A.X., Ren Z.L. Research on Current-Carrying Wear Characteristics of Friction Pair in Pantograph Catenary System. Proceedings of the 59th IEEE Holm Conference on Electrical Contacts (Holm).

[2] Wang P., Zhang H., Yin J., Xiong X., Tan C., Deng C., Yan Z. (2017). Wear and friction behaviours of copper mesh and flaky graphite-modified carbon/carbon composite for sliding contact material under electric current. Wear.

[3] Xiong X.Z., Tu C.J., Chen D., Zhang J.Q., Chen J.H. (2014). Arc erosion wear characteristics and mechanisms of pure carbon strip against copper under arcing conditions. Tribol. Lett..

[4] Ding T., Chen G., Wang X., Zhu M., Zhang W., Zhou W. (2011). Friction and wear behavior of pure carbon strip sliding against copper contact wire under AC passage at high speeds. Tribol. Int..

[5] Zhang X.J., Yang W.C., Zhang J.Y., Ge X.Y., Liu X.R., Zhan Y.Z. (2019). Multiscale graphene/carbon fiber reinforced copper matrix hybrid composites: Microstructure and properties. Mater. Sci. Eng. A.

[6] Singh M.K., Gautam R.K. (2018). Mechanical and tribological properties of plastically deformed copper metal matrix nano composite. Mater. Today Proc..

[7] Wang H., Zhang Z.-H., Hu Z.-Y., Song Q., Yin S.-P., Kang Z., Li S.-L. (2018). Improvement of interfacial interaction and mechanical properties in copper matrix composites reinforced with copper coated carbon nanotubes. Mater. Sci. Eng. A.

[8] Kalinin Y.E., Kashirin M.A., Makagonov V.A., Pankov S.Y., Sitnikov A.V. (2018). Effect of Carbon on the Electrical Properties of Copper Oxide-Based Bulk Composites. Phys. Solid State.

[9] Kováčik J., Emmer S. (2011). Thermal expansion of Cu-graphite composites: effect of copper coating. Met. Mater..

[10] Kanders U., Kanders K., Maniks J., Mitin V., Kovalenko V., Nazarovs P., Erts D. (2015). Nanoindentation response analysis of Cu-rich carbon–copper composite films deposited by PVD technique. Surf. Coat. Technol..

[11] Liu L., Zhu L.Y., Yi M.Z., Ran L.P. (2017). Effect of copper coated graphite powder content on the microstructure and properties of Cu/C composite. Carb. Tech..

[12] Samal C., Parihar J., Chaira D. (2013). The effect of milling and sintering techniques on mechanical properties of Cu–graphite metal matrix composite prepared by powder metallurgy route. J. Alloys Compd..

[13] Moghadam A.D., Omrani E., Menezes P.L., Rohatgi P.K. (2015). Mechanical and tribological properties of self-lubricating metal matrix nanocomposites reinforced by carbon nanotubes (CNTs) and grapheme—A review. Compos. Part B Eng..

[14] Nie J., Jia X., Jia C., Li Y., Zhang Y., Shi N. (2011). Friction and wear properties of copper matrix composites reinforced by tungsten-coated carbon nanotubes. Rare Met..

[15] Allabergenov B., Kim S. (2013). Investigation of electrophysical and mechanical characteristics of porous copper-carbon composite materials prepared by spark plasma sintering. Int. J. Precis. Eng. Manuf..

[16] Ma W., Lu J., Wang B. (2009). Sliding friction and wear of Cu—Graphite against 2024, AZ91D and Ti6Al4V at different speeds. Wear.

[17] Pietrzak K., Sobczak N., Chmielewski M., Homa M., Gazda A., Zybala R., Strojny-Nedza A. (2016). Effects of Carbon Allotropic Forms on Microstructure and Thermal Properties of Cu-C Composites Produced by SPS. J. Mater. Eng. Perform..

[18] Gao Q., Wu Y.Y., Zhai N., Hong Q. (2002). Conductance Research of Copper–graphite Material. Mater. Mech. Eng..

[19] Ge Y.X., Yang Z.H., Sun L.M., Zhang Y.Z., Zhang J.W. (2019). Effect of sintering temperature on properties of copper–graphite composite materials. Trans. Mater. Heat Treat..

[20] He D.H., Manory R. (2001). A novel electrical contact material with improved senlubrication for railway current collectors. Wear.

[21] Fan Y., Zhang J.S., Gao Y., Zhuang Y.H. (2006). Friction and wear characteristics of SiC and graphite hybrid reinforced copper matrix materials at high temperature. J. Tribol..

[22] Qian G., Feng Y., Chen Y.-M., Mo F., Wang Y.-Q., Liu W.-H. (2015). Effect of WS2 addition on electrical sliding wear behaviors of Cu–graphite–WS2 composites. Trans. Nonferrous Met. Soc. China.

[23] Xu W., Hu R., Li J.-S., Zhang Y.-Z., Fu H.-Z. (2012). Tribological behavior of CNTs-Cu and graphite-Cu composites with electric current. Trans. Nonferr. Met. Soc. China.

